# Biomedical Data Annotation: An OCT Imaging Case Study

**DOI:** 10.1155/2023/5747010

**Published:** 2023-08-22

**Authors:** Matthew Anderson, Salman Sadiq, Muzammil Nahaboo Solim, Hannah Barker, David H. Steel, Maged Habib, Boguslaw Obara

**Affiliations:** ^1^School of Computing, Newcastle University, Urban Sciences Building, Newcastle upon Tyne NE4 5TG, UK; ^2^Sunderland Eye Infirmary, Queen Alexandra Rd, Sunderland NE4 5TG, UK; ^3^Bioscience Institute, Newcastle University, Catherine Cookson Building, Newcastle upon Tyne NE2 4HH, UK

## Abstract

In ophthalmology, optical coherence tomography (OCT) is a widely used imaging modality, allowing visualisation of the structures of the eye with objective and quantitative cross-sectional three-dimensional (3D) volumetric scans. Due to the quantity of data generated from OCT scans and the time taken for an ophthalmologist to inspect for various disease pathology features, automated image analysis in the form of deep neural networks has seen success for the classification and segmentation of OCT layers and quantification of features. However, existing high-performance deep learning approaches rely on huge training datasets with high-quality annotations, which are challenging to obtain in many clinical applications. The collection of annotations from less experienced clinicians has the potential to alleviate time constraints from more senior clinicians, allowing faster data collection of medical image annotations; however, with less experience, there is the possibility of reduced annotation quality. In this study, we evaluate the quality of diabetic macular edema (DME) intraretinal fluid (IRF) biomarker image annotations on OCT B-scans from five clinicians with a range of experience. We also assess the effectiveness of annotating across multiple sessions following a training session led by an expert clinician. Our investigation shows a notable variance in annotation performance, with a correlation that depends on the clinician's experience with OCT image interpretation of DME, and that having multiple annotation sessions has a limited effect on the annotation quality.

## 1. Introduction

OCT is an interferometric imaging technique which can provide cross-sectional views of the subsurface microstructure of biological tissue [1]. Due to its noninvasive nature, simplicity of use, absence of ionising radiation, and high resolution, OCT has seen widespread use in diagnostic ophthalmology and has played a critical role in the diagnosis and monitoring of eye diseases such as age-related macular degeneration (AMD) and DME [2] as well as other retinal vascular diseases and macular disorders. The role of OCT analysis is also expanding in the prediction and monitoring of systemic neurodegenerative diseases. Providing high-resolution information on anatomic and structural changes within the eye, the technology can allow clinicians to detect early disease changes and monitor response to treatments, hence consequently improving patients' outcomes. With the development of spectral-domain OCT, clinicians are now able to capture denser 3D images, generating large amounts of patient-specific data. While OCT imaging holds promising benefits, the challenge of interpreting an extensive array of biomarkers using both 2D and 3D perspectives, combined with the need for comparisons to prior scans, presents difficulties. This challenge is particularly pronounced within clinics already grappling with substantial workloads. Due to this, each patient has become a “big data” challenge [3].

Automated image segmentation algorithms have emerged to provide meaningful data from medical images. These algorithms use deep learning, a subfield of machine learning that has proven successful in retinal structure segmentation in fundus [4] and OCT images [5, 6]. The use of deep learning has stimulated a proliferation in automatic assessments of various segmented features of disease pathologies within an OCT volume, particularly IRF, subretinal fluid, pigment epithelial detachment, and subretinal hyper-reflective material [7–9].

Supervised deep neural networks have achieved strong performance in medical image segmentation, in part due to high-quality annotated data [10]. However, the collection of high-quality image data annotations has many hurdles, including time and monetary costs, as well as regulatory issues [11]. To appropriately model the underlying data distribution of a given task, current supervised deep learning systems rely on labelled data. The quality of the labelled data is one of the most important aspects in achieving the desired performance for computer vision algorithms driven by deep learning. Furthermore, due to obscure boundaries of morphological features pertaining to disease pathology on medical images, annotations can suffer from inter/intraobserver variance even among specialists. As deep neural networks are vulnerable to overfitting, accepting annotations as perfectly correct and reliable may result in biased models with poor generalization [12]. This further necessitates the development of more robust annotation quality analysis approaches for the creation of more reliable and applicable datasets.

In this study, we investigated the quality of IRF annotations from multiple clinician annotators on OCT images collected from patients with DME. The main focus of this work is to explore how the quality (as evaluated through comparison of a given annotation against a ground truth) of OCT-based image annotations can vary based on clinician experience. As well as this, we explore the effect of having multiple annotation rounds on the resulting annotation quality. To evaluate the underlying variations in the annotations, image-based properties such as image noise and pixel intensity are also considered.

### 1.1. Related Work

Data quality and annotation techniques have been investigated in recent years in different fields of artificial intelligence due to their importance. McCulloh et al. [13] described numerous measures for assessing interannotator agreement, rater consistency, and distraction rate, applied to natural language processing tasks. They demonstrated improvement in annotator utilisation, interannotator agreement, and the rate of annotation through the management of the annotation process by actively monitoring several quality and schedule metrics.

For automated analysis of OCT imaging, there has been limited work in the literature investigating how annotation quality can be evaluated. Kurmann et al. [14] implemented an image annotation quality control for OCT B scans in the form of majority voting and intergrader performance evaluation using Kappa metrics, which improved the performance of a machine learning model for the classification of AMD biomarkers. Apostolopoulos et al. [15] investigated the use of automatic image enhancement on OCT B scans prior to annotating, resulting in greater annotation quality between two graders for several biomarkers.

Most investigations into how the annotator's experience can affect annotation quality are related to crowd-sourcing, which describes the use of many nonexperts for large-scale labelling and the rapid generation of labelled images for algorithm development and evaluation. Irshad et al. [16] showed that crowd-sourced nonexpert-derived evaluation metrics perform at a level similar to both research fellow and automated method-derived metrics for the relatively simple task of nucleus detection and segmentation, with the research fellow annotations showing the strongest performance for detection and the crowd-sourced scores showing the strongest performance for segmentation. They concluded that crowd-sourced image annotation is a highly scalable and effective method of obtaining nucleus annotations for large-scale projects in computational pathology.

Others have also investigated the impact of annotated data quality on algorithm performance measurements. Using multiple publicly available datasets of stationary ground-based camera data of outdoor spaces and urban scenes, Joseph et al. [17] conducted several data annotation experiments to measure interannotator agreement and consistency, as well as how the selection of ground truth impacts the perceived algorithm performance. They showed that when not monitoring the quality of annotations during the annotation process, subsequent algorithm precision can have a significant loss and that knowledge of the labelling process, training data, and ground truth data used for algorithm evaluation are fundamental components to accurately assess the trustworthiness of an AI system. Walter and Sörgel [18] identified common issues in crowd-sourced geographic data annotation, such as object misrepresentation. They demonstrated that by annotating the data numerous times and using a single shared dataset, some of these quality discrepancies can be removed.

Though there have been investigations into quality control of annotation efforts for a range of imaging modalities, to the best of our knowledge, there has been little work to show to the extent which junior clinicians can be utilised for the development of biomedical imaging annotations, and how these annotations compare to that of more experienced clinicians, especially for the case of OCT images.

## 2. Materials and Methods

### 2.1. OCT Imaging Data

The images utilised for this study were captured using the SD-OCT Heidelberg Spectralis (Heidelberg, Germany) as part of routine clinical care, using standard imaging protocol at Sunderland Eye Infirmary, UK. The dataset consists of 10 randomly selected individual OCT B scan slices from 10 patients with active DME who attended vascular clinics for treatment and follow-up. The imaging protocol used had a scan width of 15 × 30° with 19 scans (240 microns spacing) and an ART value of 9 (high speed). The individual line scans had a slight variation in size and scaling but were typically 732 by 428 pixels with a scaling of 10.5 microns per pixel on the *X* (horizontal) axis and 3.8 microns per pixel on the *Y* (vertical) axis.

### 2.2. Annotation Protocol

The annotation process took place utilising annotation software developed by Gliff [19]. The annotation tool is designed to work on any kind of imaging modality, with intuitive features of 3-step zooming, contrast adjustment of the image or the annotation, and precision annotation size to subpixel level. 10 OCT images were graded using the tool by 5 clinicians with different levels of experience in OCT interpretations. The clinicians whose annotations are used in this study are anonymised for confidentiality reasons into abbreviations of expert “Ex_*i*_” and junior “Jr_*i*_.” The clinician experience is as follows: Ex_1_ > 15 years and Ex_2_ > 15 years. Both experts are senior ophthalmology clinicians and retinal specialists. All juniors are ophthalmic trainees at different levels of their specialist training: Jr_1_ 7 years, Jr_2_ 2 years, and Jr_3_ 0 years.

A clinician ophthalmologist, Ex_1_, performed the annotation once using the platform. A second clinician ophthalmologist, Ex_2_, performed the annotations two times—once before each trainee training session (with a minimum of 1-month period between each round). Ex_2_ also conducted a training session for the three clinicians considered juniors. Training focused on understanding the tool features and identifying and defining various OCT landmarks, and the features and characteristics of IRF cysts, as well as other common OCT image defects, such as vessels and exudates shadowing or dark retinal tissue that can mimic IRF cysts. The clinicians were then presented with the 10 training OCT scans of various clarities and with a wide mix of OCT features. The 3 junior clinicians were given the task of annotating all areas of IRF cysts, with no limit on size. They were instructed to shade the entire area of IRF cysts, rather than edges only. Following the first round of annotations, the annotated images were collated and reviewed by Ex_2_. A training session was set to review the results of the 3 junior clinicians to identify any wrongly defined areas of IRF cysts and evaluate over or underestimation trends of annotation and identify correct edges of the different IRF cysts, especially in images with low-resolution or ill-defined edges with poor scan quality. The clinicians were instructed to have a month cooling period before further attempts for the second round of annotation using the same platform and taking into consideration, the training provided and remarks made to improve the accuracy of defining cysts' edges and borders. None of the clinicians had access to other clinicians' annotations to prevent any influence on results. Ground truth annotations were generated through a majority vote of clinicians Ex_1_ and Ex_2_ last round of annotations to create the most accurate representation of ground truth possible.

Despite only 10 OCT images being used for this study, 2399 IRF areas were annotated across all clinicians and annotation rounds. Furthermore, the 10 images used represent scans that cover a large range of variations in both image noise/clarity, as well as disease progression, therefore allowing a greater insight into quality variation for the clinicians under different circumstances.

### 2.3. Annotator Evaluation

#### 2.3.1. Majority Voting

**Figure 1:**
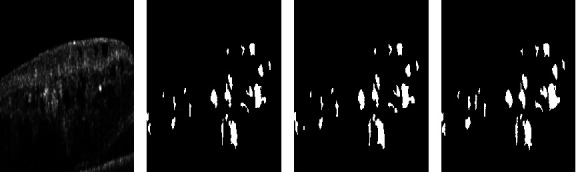
Sample cropped expert annotations with correlated OCT region and the majority voted ground truth (GT). (a) OCT. (b) GT. (c) Ex_1_. (d) Ex_2_.

We use majority voting to generate expert agreed ground-truth annotations, following the approach detailed by Kajino et al. [20].  Figure 1 shows an example of a majority vote with the correlated OCT region.

#### 2.3.2. Performance Metrics

To assess annotator performance, annotations are evaluated against the majority vote. Quantitative metrics used include intersection over union (IoU), dice similarity coefficient (DSC), true-negative rate (TNR), true-positive rate (TPR), precision, and Cohen's kappa. The metrics are calculated using the following equations:

(1) $$\begin{array}{c} IoU=\frac{TP}{TP+FP+FN}\text{,}\\ \text{DSC}=\frac{2\text{TP}}{2\text{TP}+\text{FP}+\text{FN}}\text{,}\\ \text{TNR}=\frac{\text{TN}}{\text{TN}+\text{FP}}\text{,}\\ \text{TPR}=\frac{\text{TP}}{\text{TP}+\text{FN}}\text{,}\\ \text{precision}=\frac{\text{TP}}{\text{TP}+\text{FP}}\text{,} \end{array}$$

where TP, TN, FP, and FN refer to true-positive, true-negative, false-positive, and false-negative regions, respectively. TP represents the number of pixels which are part of the region that are labelled correctly by both the annotator and the ground truth. TN represents the number of pixels which are part of the background region and labelled correctly by both the annotator and the ground truth. FP represents the number of pixels labelled as part of the region by the annotator but labelled as a part of the background by the ground truth. Finally, FN represents the number of pixels labelled as part of the background by the annotator but labelled as part of the region in the ground truth.

Cohen's kappa coefficient is used as a metric to analyse the reliability of the agreement among annotators, which has the benefit over the alternatives of accounting for the possibility of the agreement happening by chance. The coefficient ranges between 0 when the agreement is not better than chance and 1 when there is perfect agreement [21]. Kappa values are grouped as follows: *κ* ≤ 0 indicating no agreement, *κ* ≥ 0.01 and *κ* ≤ 0.20 as none to slight, *κ* ≥ 0.21 and *κ* ≤ 0.40 as fair, *κ* ≥ 0.41 and *κ* ≤ 0.60 as moderate, *κ* ≥ 0.61 and *κ* ≤ 0.80 as substantial, and *κ* ≥ 0.81 and *κ* ≤ 1.00 as almost perfect agreement. *κ* is calculated using the following equation:

(2) $$\begin{array}{c} \kappa =\frac{p_{o}-p_{e}}{1-p_{e}}\text{,} \end{array}$$

where *p*_*o*_ represents the relative observed agreement among annotators and *p*_*e*_ is the hypothetical probability of chance agreement, both calculated using the following equations:

(3) $$\begin{array}{c} p_{o}=\frac{\text{TP}+\text{TN}}{\text{TP}+\text{TN}+\text{FP}+\text{FN}}\text{,}\\ p_{e}=\frac{\left(\text{TP}+\text{FN}\right)\cdot \left(\text{TP}+\text{FP}\right)+\left(\text{FP}+\text{TN}\right)\cdot \left(\text{FN}+\text{TN}\right)}{{\left(\text{TP}+\text{TN}+\text{FP}+\text{FN}\right)}^{2}}\text{.} \end{array}$$

Gwet's AC_1_ is also calculated similarly as another interrater agreeability metric [22]. Gwet's AC_1_ tends to be less influenced by imbalanced data and prevalence than Cohen's kappa. Therefore, providing values for Gwet's AC_1_ alongside Cohen's kappa is recommended due to strong evidence to support its benefits, as detailed by Wongpakaran et al. [23]. Gwet's AC_1_ has the same structure as Cohen's kappa described in equation (2); however, the main difference lies in the calculation of *p*_*e*_:

(4) $$\begin{array}{c} {AC}_{1}=\frac{p_{o}-p_{e}}{1-p_{e}}\text{,}\\ p_{e}=p_{1}\;*p_{1}+p_{2}\;*p_{2}+p_{3}\;*p_{3}+\cdots +p_{n}*p_{n}\text{,} \end{array}$$

where *p*_1_, *p*_2_, *p*_3_,…, *p*_*n*_ are the probabilities of each category occurring for both raters and *n* is the number of categories. Therefore, in the case of binary annotations, we define *p*_*e*_ as follows:

(5) $$\begin{array}{c} p_{e}=p_{\text{positive}}\;*p_{\text{positive}}+p_{\text{negative}}*p_{\text{negative}}\text{,}\\ p_{\text{positive}}=\frac{\left(\text{TP}+\text{FP}\right)}{\left(TP+\text{TN}+\text{FP}+\text{FN}\right)}\text{,}\\ p_{\text{negative}}=\frac{\left(\text{FN}+\text{TN}\right)}{\left(\text{TP}+\text{TN}+\text{FP}+\text{FN}\right)}\text{.} \end{array}$$

### 2.4. Annotation Image Analysis

**Figure 2:**
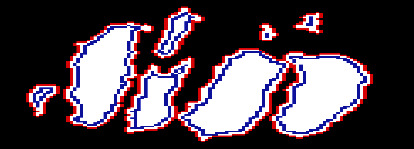
Annotated areas with highlighted outer boundary (red) and inner boundary (blue).

To better understand the underlying variance in the annotations of IRF from the annotators, image properties of the annotations against the corresponding OCT images are evaluated. IRF appears as round, minimally reflective spaces within OCT images [24]; therefore, we expect the annotations to be present in dark, rounded regions within the image. We evaluate the greyscale pixel intensity difference between the outer and inner boundaries of each annotation, with each referring to 1 pixel outside or inside the edge of the annotated region, respectively. As IRF regions are visibly darker than the surrounding tissue, this is used to gain insight into whether clinicians are over/undersegmenting. Where the IRF regions have normalised greyscale pixel intensity values closer to 0 (black) and surrounding tissue closer to 0.5 (grey), a larger difference in the pixel intensity between outer/inner annotation boundaries indicates a more accurate annotation.  Figure 2 shows the annotation boundaries for some annotated areas.

**Figure 3:**
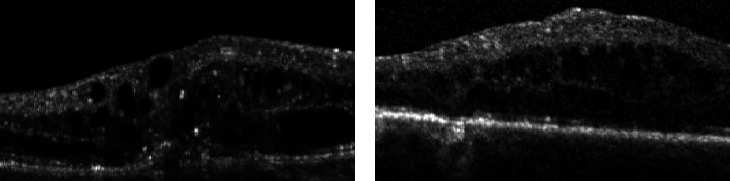
OCT images of DME with a low and high noise estimate. (a) Low *λ* = 4.88. (b) High *λ* = 80.76.

Due to OCT images suffering from noise defects, annotator performance for different noise levels is evaluated to determine the impact of noise on the annotations. As the speckle noise present in OCT follows a Poisson distribution [25], the shape parameter *λ* for each OCT image is estimated using the approach detailed by Laligant et al. [26]. Each of the 10 OCT images used is then assigned to groupings of low noise (*λ* ≥ 0, *λ* ≤ 20), medium noise (*λ* ≥ 20, *λ* ≤ 40), and high noise (*λ* ≥ 40). The subsequent distribution had 5 OCT images designated as low noise, 3 as medium noise, and 2 as high noise.  Figure 3 shows OCT images from our dataset with a low and high noise estimate.

**Figure 4:**
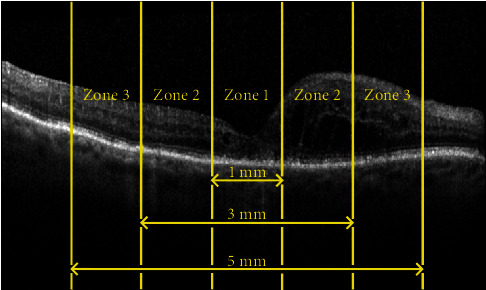
Zone locations within an OCT image.

Finally, we investigate whether the quality of the annotation varies based on the location of the perceived IRF biomarker on the OCT image. Three zones are established at locations moving outward in each direction from the centre of the OCT image, with a vertical crop at 0.5 mm, 0.5 mm–1.5 mm, and 1.5 mm–3 mm.  Figure 4 illustrates these zones on an OCT image from our dataset. Recent research work has shown variable correlations between fluctuations in fluid volumes in different central zones of the OCT scans and visual outcomes after treatment, as well as differences between 2D area measurements and estimates of fluid versus 3D volumetric measurements. It is, therefore, prudent to assess and compare the annotation accuracy for these different zones for future interpretation of results.

## 3. Results

### 3.1. Clinician Grading of IRF

**Figure 5:**
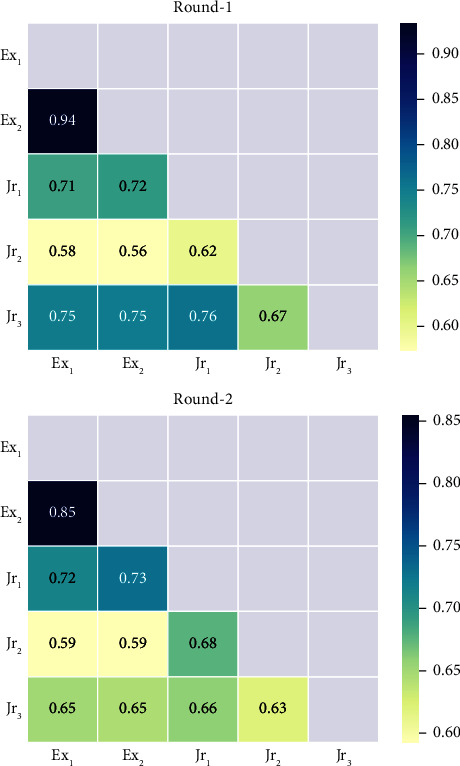
Intergrader Cohen's kappa values for round-1 and round-2 annotations. Annotations for Ex_1_ round-2 are the same as round-1 due to limited data.

**Figure 6:**
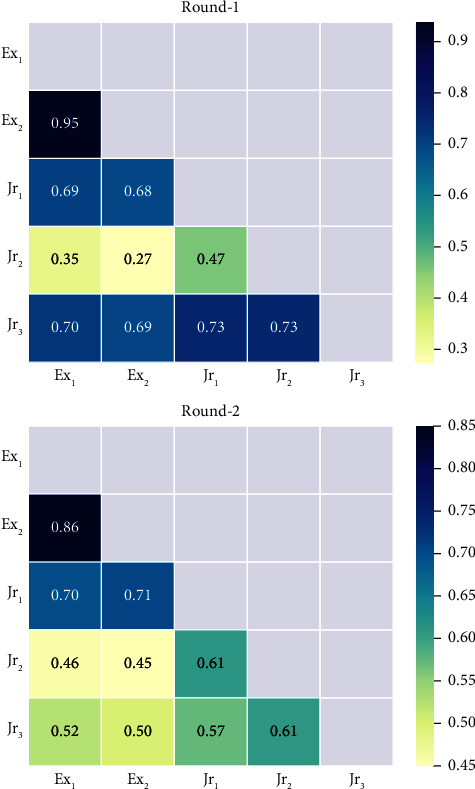
Intergrader Gwet's AC_1_ values for round-1 and round-2 annotations. Annotations for Ex_1_ round-2 are the same as round-1 due to limited data.

The mean *κ* value for the IRF annotation agreement across both round-1 and round-2 annotations was 0.68, with minimum and maximum scores of 0.56 and 0.94, respectively. The mean *κ* value for round-2 was lower than that for round-1 (*κ*_round−2_=0.68 < *κ*_round−1_=0.71). The highest agreement was observed for expert clinician's Ex_1_ and Ex_2_ on round-1 annotations (*κ*=0.94), indicating almost perfect agreement, and the lowest agreement was seen for clinician's Ex_2_ and Jr_2_ on round-1 annotations (*κ*=0.56), indicating moderate agreement.  Figure 5 shows the relations of *κ* between each pair of graders for the annotations of round-1 and round-2. In comparison, the mean AC_1_ value for round-2 was also found to be lower than that for round-1 (AC_1_round−2__=0.60 < AC_1_round−1__=0.63). The same maximum and minimum agreement values between pairs were found as with previously stated Cohen's kappa pairs.  Figure 6 shows the relations of the AC_1_ value between each pair of graders for the annotations of round-1 and round-2.

**Figure 7:**
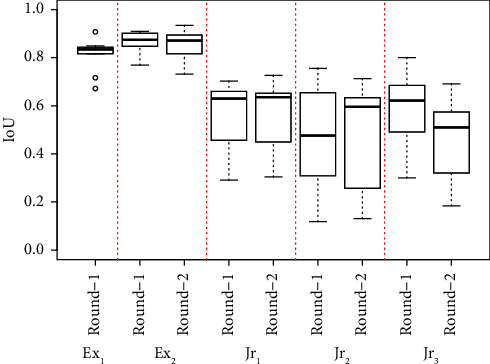
Grading performance of clinicians against the ground truth for each round of annotations.

Tables 1 and 2 describe the performance of each clinician grader against the majority vote, with metrics averaged across all annotations. Columns “# of annotations” within each table refer to the number of annotated fluid areas within the images.  Figure 7 shows the distribution of IoU scores for each clinician's annotation rounds. Performance metrics had limited improvement in the second round of annotations for the 4 annotators with a second round, where 9 of 20 metric values across IoU, DSC, TNR, TPR, and precision increased, with 2 remaining the same and 9 decreasing.

### 3.2. Annotation Image Region Properties

**Figure 8:**
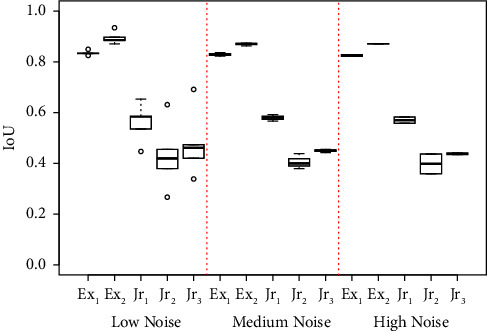
Grading performance for each clinician's last round of annotations for OCT images at each estimated noise level.

The variance of the IoU values for each clinician's last round of annotations grouped into different noise levels can be observed in Figure 8. Image noise did not have a significantly strong correlation to annotator performance, with mean IoU for expert and junior clinicians in low noise images of 0.8648 and 0.4304, respectively, whilst in high noise expert and junior, mean IoU is 0.8479 and 0.4687, respectively.

**Figure 9:**
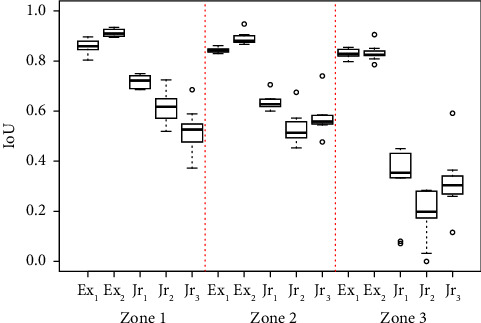
Grading performance for each clinician's last round of annotations in each defined zone across 10 annotated OCT images.

Figure 9 shows the variance of the IoU values across the three zones previously defined for each clinician's last round of annotations. A relationship between the location of the annotation and the performance of the annotator can be seen, particularly for juniors with a large decrease in mean IoU farther from the centre of the OCT image. The mean IoU for expert and junior clinicians in zone 1 is 0.8857 and 0.6179, respectively, whilst in zone 3, expert and junior mean IoU reduces to 0.8305 and 0.2782, respectively.

**Figure 10:**
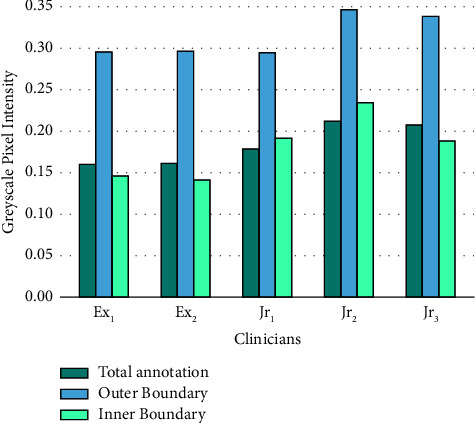
Mean normalised pixel intensities of all annotations and outer/inner boundaries.

Figure 10 depicts the mean normalised greyscale pixel intensity values for each clinician's last round of annotations for the 10 OCT images. A larger difference between the expert's outer and inner annotation boundaries can be seen with a mean difference of 0.1522, in comparison with the juniors which have a mean difference of 0.1216. Furthermore, the greyscale pixel intensity of the entire area of annotations shows that on average, junior annotations have higher greyscale intensity annotations than the experts. Both factors suggest that when annotating the IRF areas, junior annotations tend to be slightly oversegmented into the surrounding retinal tissue.

## 4. Discussion

Our experimental results show that the quality of annotations can have a notable range based on clinician experience. Differences in the annotation quality between the clinicians used within this study could be attributed to human-centric limitations during the manual annotation process such as clinical experience, lack of annotator focus, and distractions.

Inter-rater agreeability analysis found that agreement as evaluated through Gwet's AC_1_ is lower than that of Cohen's kappa. This could be attributed to the calculation of Gwet's AC_1_, which takes into account the prevalence of categories and adjusts for imbalanced data. As our data are imbalanced, with a majority of pixels in each annotation being background, this suggests the clinicians agree on the more prevalent category (background), but disagree more on the less prevalent category (IRF), which can be expected. Despite the slight disparity in the absolute values of the two metrics, they reveal comparable trends of agreeability between clinicians, where the pairs of clinicians with the highest and lowest agreement metrics remain consistent across both measures.

A unique feature detailed within our investigation was an image analysis-based method to evaluate the variance of the image properties of the annotations. This showed that expert clinicians have precise annotations which are more contained to the IRF areas, whilst less experienced clinicians have larger and less precise annotations which oversegment. Furthermore, we found the noise present in the OCT image not to have a significant effect on annotation quality; however, annotations farther away from the centre of the scan have a significant reduction in quality for junior clinicians. Our approach of having a clinician annotation of the same image over multiple rounds showed little improvement in the quality of the annotations against the ground truth. This could be due to the training approach undertaken not being effective, or that such biomarker identification on OCT images takes many years of clinical experience to improve upon.

We have also shown that the identification of DME biomarkers in OCT images is a challenging task even for clinicians with decades of clinical experience. Whilst this variation might be attributed to a variety of reasons, including adherence to the biomarker specification, size, extent of the biomarker in the picture, or image quality, the intergrader agreeability between experts was high.

There are some limitations to the methods we have used. This investigation was carried out with very limited data; therefore, the results may be skewed to this small sample. As well as this, with only 2 experts to create the majority vote, when evaluating the performance of the data used to create the majority vote, there will be some bias. This could be prevented by having a minimum of 3 expert clinicians included and using a leave-out approach where performance metrics and intergrader agreeability are also evaluated for a majority vote in which their annotations are not included, as shown in the investigation by Kurmann et al. [14]. Finally, it would be beneficial to include annotations of other biomarkers to determine how the quality of the annotations may vary depending on the biomarker, as only IRF annotations were considered in this study.

## 5. Conclusion

In this work, we investigated the variance in annotation quality on OCT image annotations from multiple annotators with different levels of experience. Based on intergrader agreeability metrics, we observed that expert clinicians have an almost perfect agreement with a moderate-substantial agreement with junior clinicians, whilst juniors have substantial agreement with each other. It was found that having junior clinicians annotate over multiple training rounds had a limited improvement in quality. Through an image-based analysis, image noise had little effect on annotation quality; however, annotations farther from the centre of the image have substantially reduced quality for junior clinician annotations. Furthermore, it was found through analysis of annotation boundaries that expert clinician annotations were contained to the darker IRF areas, whereas juniors tend to oversegment into surrounding retinal tissue.

This study has shown the extent to which annotations from annotators with different levels of subject experience can vary. These findings suggest that the use of less experienced clinicians for image annotation may not be a reliable solution for improving the efficiency of data collection in ophthalmology. It may be necessary to invest in more comprehensive training programs or to prioritize the involvement of experienced clinicians in the annotation process to ensure the reliability and accuracy of automated image analysis using deep learning approaches that rely upon these annotations.

## Figures and Tables

**Table 1:** Annotator assessment on IRF round-1 annotations measured against ground truth.

Clinician	DSC	TNR	TPR	Precision	# of annotated areas
Ex_1_	0.9078	1	0.8312	1	411
Ex_2_	0.9336	0.9996	0.8983	0.9719	285
Jr_1_	0.7195	0.9981	0.6321	0.8451	219
Jr_2_	0.5527	0.9993	0.3943	0.9456	254
Jr_3_	0.7359	0.9993	0.6039	0.9491	307

**Table 2:** Annotator assessment on IRF round-2 annotations measured against ground truth.

Clinician	DSC	TNR	TPR	Precision	# of annotated areas
Ex_2_	0.9375	1	0.8825	1	285
Jr_1_	0.7235	0.9981	0.6373	0.8435	191
Jr_2_	0.5827	0.9979	0.4575	0.8291	111
Jr_3_	0.6267	0.9993	0.4725	0.9482	336

## Data Availability

The data used to support the findings of this study are available from the corresponding author upon request.
